# Discovery of *cis*-elements between *sorghum *and rice using co-expression and evolutionary conservation

**DOI:** 10.1186/1471-2164-10-284

**Published:** 2009-06-26

**Authors:** Xi Wang, Georg Haberer, Klaus FX Mayer

**Affiliations:** 1 MIPS/IBIS Institute of Bioinformatics and System Biology, Helmholtz Center Munich, D-85764 Neuherberg, Germany

## Abstract

**Background:**

The spatiotemporal regulation of gene expression largely depends on the presence and absence of *cis*-regulatory sites in the promoter. In the economically highly important grass family, our knowledge of transcription factor binding sites and transcriptional networks is still very limited. With the completion of the sorghum genome and the available rice genome sequence, comparative promoter analyses now allow genome-scale detection of conserved *cis*-elements.

**Results:**

In this study, we identified thousands of phylogenetic footprints conserved between orthologous rice and sorghum upstream regions that are supported by co-expression information derived from three different rice expression data sets. In a complementary approach, *cis*-motifs were discovered by their highly conserved co-occurrence in syntenic promoter pairs. Sequence conservation and matches to known plant motifs support our findings. Expression similarities of gene pairs positively correlate with the number of motifs that are shared by gene pairs and corroborate the importance of similar promoter architectures for concerted regulation. This strongly suggests that these motifs function in the regulation of transcript levels in rice and, presumably also in sorghum.

**Conclusion:**

Our work provides the first large-scale collection of *cis*-elements for rice and sorghum and can serve as a paradigm for *cis*-element analysis through comparative genomics in grasses in general.

## Background

In higher eucaryotes, gene transcription is controlled by a variety of mechanisms such as chromatin modifications or degradation via complementary miRNAs. Gene promoters and their *cis*-regulatory element composition, however, are the initial checkpoints for transcriptional gene activities and define the potential spatiotemporal expression of a gene. Among other aspects, knowledge of the elementary functional units – transcription factor binding sites – is a prerequisite to understanding regulation of individual genes and their embedding into regulatory networks.

Numerous approaches, both experimental and *in silico*, have been developed to uncover *cis*-regulatory elements [1,2]. Chromatin immuno-precipitation combined with microarrays/ChIP-on-chip provides direct experimental evidence for Protein-DNA interactions on genome-scale and is a powerful approach [3]. Yet, ChIP-on-chip is currently not easily applicable in many higher eucaryotes [4]. Other established experimental methods such as staggered promoter deletions or DNAseI footprints provide high-resolution views of single promoters but are infeasible for large-scale analysis. To overcome experimental limitations, computational methods have been developed as time- and cost-effective complements for large-scale motif discovery. These include mapping of known as well as detection of *de novo *motifs, e.g. [5-8]. In general, two types of data set are used for motif searches as sources of information: a group of functionally related, e.g. co-expressed, genes and orthologous promoter sequences. In the first case, candidate motifs are expected to be enriched compared to a statistical background model. Hence, they can be detected by their over-representation in the respective gene group. In the latter it is expected that non-functional regions will be considerably more diversified compared to functional *cis*-elements. In a widely applied approach, candidate sites emerge as conserved patterns or phylogenetic footprints from (local) alignments between evolutionary related sequences. Besides the use of single informative sources, several tools have been developed that combine conservation and co-expression information [9-11]. We applied PhyloCon in an earlier analysis [9]. PhyloCon detects motifs in data sets for which promoter sequences of genes co-expressed in one species are complemented with orthologous promoter sequences of one or more related species. In the first step, motif discovery is undertaken between orthologous sequences and initial motifs are generated from local alignments. In the second step, expression data are used to define groups of genes co-expressed in one of the species. Subsequently comparing and merging initial profiles between co-expression groups iteratively refines motifs. The combined application of two sources of information has been demonstrated to provide increased predictive power compared to approaches using only one source, e.g. overrepresentation [9,12].

In contrast to motif discovery from a confined or user-selected set of genes, network-level conservation detects globally conserved motifs from comparison between two genomes. Functional motifs are identified by their unusually high retention in orthologous promoter pairs in comparison to those anticipated from single genome frequencies. An alignment-free implementation of the network-level conservation principle, FASTCOMPARE, has been successfully employed to motif discovery in yeast, nematodes, fruit flies and humans [13]. In our study we adopted FASTCOMPARE to study network-level conservation in sorghum and rice.

A large number of *in silico *studies to detect de novo *cis*-regulatory elements have been reported for the bakers yeast *Saccharomyces cerevisiae *and some of its relatives [14,15]. In yeast, evaluation of biologically meaningful motifs is supported by a plethora of experimentally verified motifs as well as genome-wide ChIP-on-chip studies for transcriptional binding sites. Recently, progress in genome projects of higher eucaryotes, e.g. vertebrates and the genus Drosophila, has boosted motif discovery and our understanding of regulatory networks in these organisms [16]. In higher plants, however, thus far the lack of sequences of evolutionarily closely related plant genomes has restricted large-scale analysis mainly to dicotyledonous plants like the model system *Arabidopsis thaliana *[17,18]. For the economically and agriculturally highly important monocotyledonous plant genomes, however, until recently only the rice genome sequence was available. This limited comparative genomics approaches to a few hundred gene promoters for which orthologs in monocots have been described and analyzed [19]. With the completion and availability of the sorghum genome this limitation has now been overcome and we are now in a position to undertake genome-scale comparative studies between evolutionarily related monocotyledonous genomes.

Sorghum and rice belong to two different grass subfamilies, the *Panicoideae *and *Bambusoideae*, respectively that diverged approximately 60 million years ago [20]. Though genome sizes differ twofold, gene number and order are similar: about 60% of sorghum genes are located in syntenic regions to rice and orthologous relationships are well established by genetic markers as well as whole genome comparisons [20,21]. In addition, transcriptome data for rice that monitor genome-wide expression levels of many thousands rice genes have become available in recent years [22-24]. This now, for the first time, allows us to analyze conserved sequence elements on a genome scale and to detect candidates for transcription factor binding sites between monocotyledonous species.

In this study, candidate *cis*-regulatory elements in rice and sorghum have been elucidated by two complementary approaches. Firstly, we derived transcriptional networks in rice from correlation matrices of three independent rice expression data sets. Groups of co-expressed rice genes are obtained as maximal cliques of these networks and each gene of a clique is complemented by its sorghum ortholog. PhyloCon was applied to this data set to detect motifs in upstream sequences that are both overrepresented in co-expressed genes and conserved between orthologs. In a complementary approach, candidate motifs were identified by their preferred genome-wide conservation between syntenic promoters following the "network-level conservation" approach of Elemento and Tavazoie [13]. The numbers of motifs two genes have in common correlate with their degree of co-expression. Both methods provide the first large-scale collection of *cis*-elements for rice and sorghum and indicate promising approaches for *cis*-element discovery in grasses in general.

## Results

### Preparation of input data set

PhyloCon as well as network-level conservation use evolutionary conserved footprints for motif discovery. We identified orthologs between *Sorghum bicolor *and the genome of *Oryza sativa ssp. japonica *from syntenic regions of the two species [20,25]. Tandem duplications of genes frequently occur in plant genomes and typically comprise approximately one fifth of all genes [25,26]. To avoid complications by tandem duplications, we selected only gene pairs from syntenic regions that were detected as bidirectional best Blastp hits. In total, 15,773 orthologous gene pairs were identified. Comparison of upstream regions that have been deduced from incorrect gene starts can strongly impair motif discovery between orthologs. Thus, we restricted our analysis to those 12,129 gene pairs that fulfilled our stringent criteria for aligned orthologous N-termini (see Methods).

### Detection of locally overrepresented motifs: PhyloCon analysis

**Figure 1:**
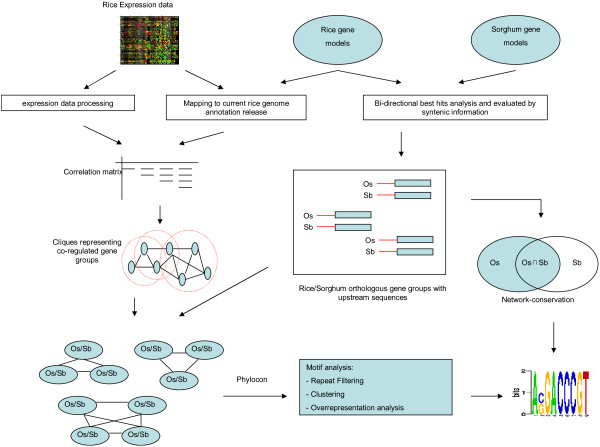
**Workflow of cis-element discovery in rice and sorghum**. Two complementary approaches have been employed for motif discovery. Starting from orthologous gene pairs between rice and sorghum, orthologous upstream sequences have been isolated. These sequences were used to identify motifs with a high conservation rate between syntenic pairs compared to their single genome frequencies (network-level conservation approach). For PhyloCon, orthologous pairs that were supported by co-expression were combined to orthologous groups and were subjected to a PhyloCon analysis. Motifs were consecutively filtered for repeats and merged by clustering. Statistical significance has been re-evaluated for each of the clustered motifs. For further details, see text and Methods.

PhyloCon analysis is based on the comparison of conserved sequence profiles from orthologous pairs that are joined via co-expression of genes from a reference genome. In this study co-expressed genes in rice were deduced from three different whole-genome expression data sets: MPSS data for several tissues and abiotic stresses [23], and two oligonucleotide array experiments covering developmental stages, various tissues [22] as well as drought and salt stress conditions [24]. In the following, we refer to the array experiments measuring developmental stages and tissues as YALE-1, while the array describing expression data for stress conditions is termed YALE-2. In total, the experiments comprise 213 individual measurements or arrays, respectively. Figure 1 shows an overview of the motif discovery scheme applied in this study.

As it is highly problematic to combine expression data derived from different platforms, functional groups were identified separately for each of the three expression sets. Firstly, we assigned MPSS tags as well as 70 mers of the oligonucleotide arrays to the current rice RAP2 gene predictions and selected genes that show significant expression levels (see Methods). Of the RAP2 gene models, 19,396 had an MPSS tag that unambiguously mapped to one gene model. The oligonucleotide arrays contain a total of 58,404 70 mers of which 27,887 had a unique match to the RAP2 gene annotation. For the YALE-1 experiment, we obtained significant measurements for 13,904 genes while 20,633 genes showed reliable expressions in the YALE-2 arrays (see Methods).

To analyze for co-expressed genes we calculated an all against all Pearson correlation matrix. For each expression set, co-expressed genes were defined as pairs whose Pearson correlation exceeded the 99%-quantile of the background distribution of all correlation coefficients. Background and quantiles were estimated from the all-against all Pearson correlation matrix. Next, an undirected graph with nodes representing genes and edges between them if genes were co-expressed, was constructed. From this graph, co-expressed gene groups were extracted as maximal cliques for each node. To avoid clusters with broad or unspecific expression patterns, we restricted our analysis to nodes with ≤ 100 edges. After filtering for orthologs with congruent N-terminal alignments we determined 4,683 cliques and 6,667 gene pairs for the MPSS data, and for YALE-1 and YALE-2 4263 cliques with 4,395 pairs, and 2,185 cliques with 2,379 pairs. The resulting co-expression groups were subjected to a PhyloCon analysis. Initially, we detected 5,337, 14,754 and 17,068 position specific scoring matrices (PSSM) from the YALE-2, YALE-1 and MPSS derived cliques, respectively. After filtering for simple repeats, the expected frequency in rice upstream regions was determined for the detected PSSM's. For each co-expression group we tested whether the respective PSSMs were statistically overrepresented (see Methods). To obtain a set of non-redundant motifs for each data set, similar PSSMs were subsequently merged to one motif by hierarchical clustering (see Methods). As regrouping of PSSMs to one motif can potentially alter its significance we retested statistical over-representation of the newly formed motifs as described above. This procedure resulted in 1,622 MPSS motifs, 1,500 YALE-1 motifs and 866 motifs from the YALE-2 data set. A list of sites supported both by sequence conservation as well as co-expression is provided (see additional files 1, 2, 3, 4, 5 &6).

### Detection of globally overrepresented motifs: Network-level conservation

PhyloCon deduces motifs from a well-defined subset of co-expressed genes and their orthologs. In contrast, network-level conservation finds motifs by their global, i.e. genome-wide high conservation rate within syntenic promoters. Our approach followed the FASTCOMPARE method as described in Elemento and Tavazoie with some modifications [13,27]. Briefly, we investigated a k-mer search space of 6 mers up to 9 mers. As many transcription factor binding sites tolerate a certain degree of sequence degeneracy, we allowed for up to three variable positions within the k-mers. For exact words, a full enumeration of all possible k-mers was analyzed. For searches allowing variable positions we applied time-effective heuristics. In addition, we included symmetric dyad motifs of the general format X{2,4}-N{2-16}-X{2,4} to cover these binding site patterns. We also tested by a heuristic technique whether specific nucleotides at initially unspecified positions within the spacer of a dyad could result in higher scoring motifs.

The number of occurrences of k-mers per gene has been determined for 2 kb upstream regions of the 12,129 syntenic gene pairs separately for both sorghum and rice genes. To score k-mers, the expected overlap for co-occurrence in syntenic pairs were calculated from single genome frequencies. Ratios of the observed and expected number of co-occurrences were normalized and transformed to z-scores. Motifs with an over-representation of two standard deviations above the mean (z-score ≥ 2) were selected as candidate motifs. This role was also supported by their unusually high degree of global conservation. Of these candidate motifs, many motifs are size variants of one core motif. For example, one of our motifs, AACCCTA, resembles some previously described plant telomere repeats (AAACCCT)n and is highly similar to the telo-box that has been identified in Arabidopsis thaliana as a *cis*-regulatory element active in meristematic cells [28,29]. Besides the sequence of this telo-box like motif, several size and degeneracy variants (e.g. AAACCC, AACCCTAG) were also highly overrepresented in our dataset. In order to deduce a non-redundant list of significant motifs, only the highest scoring k-mers were retained from size variants. Applying this motif clustering schema, 3,806 k-mers (including 457 dyad motifs) were recovered for further analysis (Table 1, Additional files 7, 8). As described previously, initial dyad motifs were allowed to converge to higher scoring, more specific motifs. We detected many cases for which a large fraction of spacer positions converted to specific letters (Table 2). Several dozens of these motifs show a very rare occurrence (3 ≤ n < 10) in the 12,129 syntenic upstream sequences of rice and sorghum but a very large fraction of occurrences in single species are conserved between syntenic pairs (Table 2). Long and unusually highly conserved motifs may provide a highly specific site or several binding sites in close proximity to ensure a coherent regulation of the respective genes, at least in one biological process or response. Co-functionality of genes sharing such sites is supported by several of our gene groups. The motif CACGNGNTTTGAC is conserved in two WRKY transcription factors and a seven-helix transmembrane protein homolog to the Mlo1 gene from barley. WRKY transcription factors as well as the Mlo1 gene have been experimentally linked to primary pathogen responses [30,31]. In another group, histones H2A and H2B as well as a high mobility group I/Y-2 are present. All these proteins are known to build or dynamically interact with chromatin structures. For the motif GCTCTNCNCNAAGA, conserved occurrences are found for enzymes of the phenylpropanoid- and lignin metabolism, two hydroxyanthranilate-hydroxycinnamoyltransferases and a ferulate-5-hydroxylase.

**Table 1:** Sample of rice motifs detected by network-level conservation.

Motif	#rice	#sorghum	Exp.	Obs.	Z-score	Known sites	Motif Name
G:A:C:C:G:T:T:A:C	20	26	0.04	5	20.1384		

G:A:G:T:A:A:C:G:C	20	16	0.03	3	19.6146		

C:C:C:C:G:A:T:A	55	63	0.29	4	13.4851		

G:C:G:G:G:A:A:A	177	196	2.86	22	6.5334	G:C:G:G:G:A:A:A	re2f-1 element

C:C:T:T:A:T:C:C	390	315	10.13	75	6.2174	C:T:T:A:T:C:C	GATA/SBX element

C:C:G:G:G:T:AG:A:T	48	26	0.1	3	6.1608		

C:T:A:C:G:C:G	434	457	16.35	42	6.1212		

A:C:G:C:G:T:G:T:C	37	31	0.09	3	4.8835	A:A:C:G:C:G:T:G:T:C	CE3 (Coupling element)

C:A:C:G:T:G:A	950	873	68.38	156	4.7977	C:A:C:G:T:G	G-box

G:G:A:C:G:T:C:A	116	104	0.99	6	4.7054	A:C:G:T:C:A	hexamer motif

T:T:A:A:T:G:CG:C:G	95	53	0.42	9	4.2548	T:T:A:A:T:G:G	Target of WUS

C:C:A:C:G:T:G	1577	1099	142.89	308	4.2168	C:C:A:C:G:T:G:G	G-box

G:T:A:C:G:T	2788	2859	657.18	874	3.9756	G:T:A:C:G:T:G	ACGT motif

A:C:C:G:A:C:G	880	800	58.04	117	3.5723	A:C:C:G:A:C	DRE

C:G:C:A:T:A:T:C	129	96	1.02	5	3.4547	C:A:T:A:T:C	I-Box

A:C:G:T:G:G:C	1408	1006	116.78	231	3.3984	A:C:G:T:G:G:C:G	ABRE

G:C:A:A:C:G:T:G:A	49	44	0.18	4	3.2268	C:A:A:C:G:T:G	OsBP-5 binding site

A:C:C:G:A:C:A:T:T	45	48	0.18	4	3.2193		

A:A:C:C:G:A:C	714	715	42.09	79	2.9321	A:C:C:G:A:C	DRE

T:T:T:C:C:C:G:C	248	244	4.99	33	2.9232	T:T:T:C:C:C:G:C	E2F binding site

G:G:G:C:C:C	3813	3394	1066	1472	2.8948	G:G:G:C:C:C	ERE

T:A:G:C:C:G:C:C:T	56	55	0.25	5	2.7213	A:G:C:C:G:C:C	AGC box

G:C:G:G:T:AT:A:T:T	53	44	0.19	3	2.7055	G:C:G:G:T:A:A:T:T	GT2 binding site

G:C:A:C:G:T:G:G	258	219	4.66	19	2.5531	C:A:C:G:T:G:G	G-box plus G

T:A:A:C:C:C:T:A	432	337	12	48	2.4654	A:A:C:C:C:T:A	Telo-box

A:C:T:T:T:G:C:G	114	134	1.26	5	2.4333	A:C:T:T:T:G	T-box

T:A:C:G:T:A:C	1119	1210	111.63	194	2.2906	T:A:C:G:T:A	A-box

T:A:G:C:C:G:C:C:A	68	62	0.35	6	2.285	A:G:C:C:G:C:C	AGC box

C:A:A:C:G:T:G:G	249	178	3.65	14	2.2805	C:A:A:C:G:T:G	OsBP-5 binding site

G:G:G:T:A:A:T:CT:G	62	44	0.22	3	2.128	G:G:T:A:A:T:T	GT2 binding site

Table gives motif examples with a high conservation rate in rice-sorghum orthologs. A full list of detected motifs is provided in additional files 7 and 8. The number of genes (out of 12,129 syntenic genes) in rice and sorghum containing the respective motif in their upstream sequence are shown in the columns '#rice' and '#sorghum', respectively. The following columns show the number of expected and observed co-occurrences in syntenic pairs, as well as the z-score. Matches to known sites/motifs are indicated in the last two columns.

**Table 2:** Sample of rice long specific motifs detected by network-level conservation.

Motif	#rice	#sorghum	#common	Zscore
T:A:G:C:G:C:G:T:C:T:G:A:C:T:T:C:A:G:A:T:C:A:G:A:A	5	3	3	18.51

G:G:A:C:C:A:G:A:N:C:N:T:N:A:N:T:C:T:G:G:C:G:C:C:T:T:A:G:A:C:C:A	4	3	3	16.27

A:C:G:C:G:G:C:G:A:A:G:C:A	3	3	3	16.04

G:G:A:A:T:G:C:N:G:A:A:A:G:A:T:G:T:G	3	3	3	14.41

T:T:C:T:N:N:G:N:N:G:N:N:T:T:C:C:T:C:T:A:C:T:G:G:T:T:N:T:A:N:G:T:C:T:T:C:T:C:A	4	4	4	11.11

C:A:T:G:T:G:C:N:N:G:C:A:C:G:T	4	4	3	8.3

G:T:G:G:G:A:T:T:T:G:A:A:C:C:C:A:C:G:C:C:C:T	4	4	3	8.02

C:C:C:T:T:T:N:G:G:A:C:C:A	5	5	3	7.73

G:A:A:T:C:C:C:C:N:C:C:A:A:A:A	5	4	3	6.09

A:A:C:C:C:T:A:G:A:T:C:T:C	4	6	3	5.93

A:G:A:T:C:C:A:G:A:T:C:C	7	8	4	4.54

C:A:C:G:T:C:A:N:C:G:A:T:C:C:G	9	7	6	4.16

C:C:A:C:G:T:N:A:N:N:G:A:T:C:C:G:C	7	6	5	4.11

C:C:G:A:G:C:C:A:A:A:A	10	12	3	2.15

Examples for long specific motifs are shown that emerged from dyad motifs with initially unspecified spacer sequences (denoted as N). Note that many motifs have a low occurrence rate in rice and sorghum; however, most or all occurrences are conserved between orthologous pairs.

### Validations of detected motifs

To validate our motifs derived from network-level conservation and PhyloCon analysis, we compared our findings to previous reports on rice. The PLACE and TRANSFAC databases were searched for matches between our motifs and known sites. PLACE and TRANSFAC currently contain 74 and 55 sequences of rice transcription factor binding sites, respectively [32,6]. However, many sites are redundant between the databases (or even within one database) or represent binding site variations. Hence, the exact number of different motifs is difficult to assess. In our search we also included motifs originally described in other plant species as cross-conservation has been reported for functional *cis*-elements [33]. Using literature searches as well as public databases containing known rice binding sites, we found 559 of our motifs matching 43 known regulatory sites out of 96 distinct sequences in both databases (see Table 1, 2 and Additional files 7, 8). In addition, motifs extracted from literature searches but not present in databases have been detected, for instance a perfect match to the ethylene response element GGGCCC and motifs highly similar to the telo-box AAACCCTA reported in *Arabidopsis thaliana *(see above).

**Figure 2:**
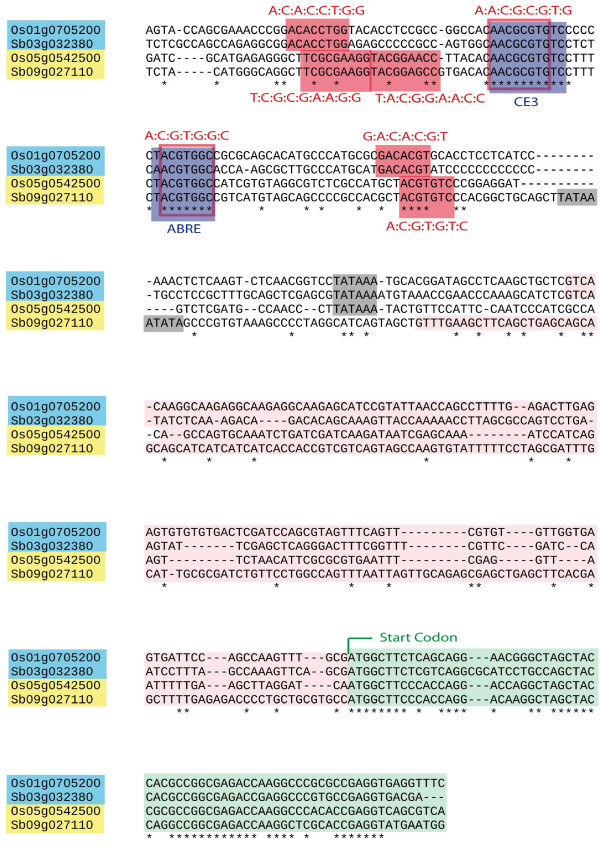
**Co-conservation of two ABA-response elements in LEA promoters**. The alignment of two rice upstream sequences with their respective sorghum syntenic partners is shown. Pairs are marked as light blue and yellow frames. The rice genes Os01g0705200 and Os05g0542500 have been annotated as late embryogenesis abundant (LEA) proteins. Two known ABA response elements, ABRE and CE3 (dark blue frames), are highly conserved in position and inter-motif distance between all four promoters. Functionality of a similar motif arrangement has been reported for other ABA responsive rice genes [34]. Additional motifs that are conserved between a rice-sorghum pair but not in all four promoters are shown as red frames and may indicate different responses between the pairs. Gray frames, pink and light green fragment depict potential TATA box, annotated transcript and annotated coding region, respectively.

Figure 2 depicts two known cis-elements conferring transcriptional ABA responses, the G-box related ABA response element (ABRE) and the coupling element 3 (CE3) as an example of co-conservation [34]. The full list of network-level conserved motifs is provided as additional files (see additional files 7, 8).

**Figure 3:**
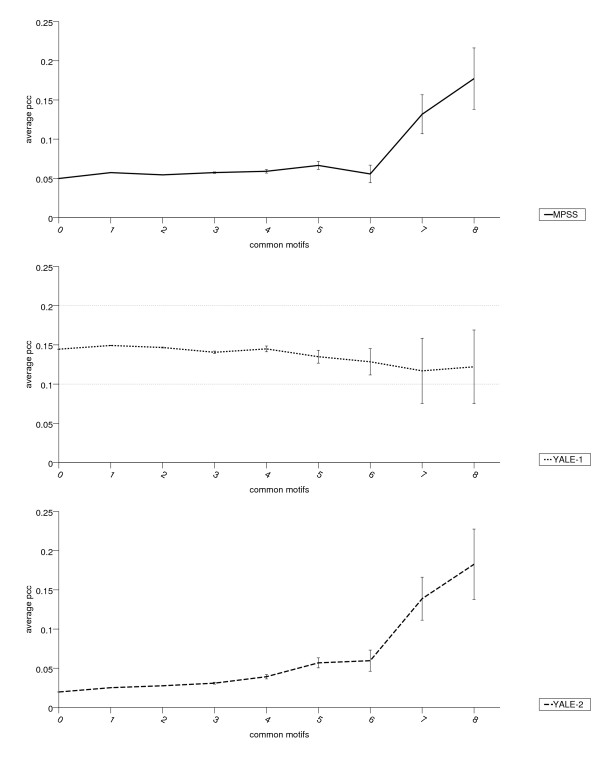
**Dependency between number of shared motifs and expression similarity**. On the x-axis, the shared number of network-level conserved motifs for all pairwise comparisons of the 12,129 rice genes are shown. Common motif numbers are binned into 0, 1, 2.. up to 8 or more motifs shared between two rice upstream sequences. Expression similarities are provided on the y-axis as mean Pearson correlation coefficients with error bars for each bin. Figure 3 show results for expression data sets of MPSS, YALE-1 and YALE-2. Positive trends in MPSS and YALE-2 are significant.

Responses to environmental changes and expression patterns in higher eucaryotes frequently result from the combinatorial actions of two or more transcription factors that bind to several distinct *cis*-elements within a promoter [35]. For functional elements that control transcriptional activities, it is therefore expected that the number of shared elements of a gene pair will correlate with its expression similarity. For rice promoter pairs we analyzed the relation of co-occurrence of motifs detected by network-level conservation and their expression congruency. Expression similarity between a rice gene pair was measured by the Pearson correlation coefficient. To determine particular candidate motifs for a rice gene, all significant motifs were selected that were present in both upstream sequences of a rice gene and its respective syntenic sorghum partner. Pairs were binned according to the number of motifs they have in common, and for each bin we determined the mean Pearson correlation from its members. As shown in Figure 3, a positive association between the number of shared motifs and the Pearson correlation coefficient for MPSS and YALE2 but not for YALE1 data was detected. Chi-square tests show significant deviations from independency (df = 72; chi-square sums 3128 and 6046 for MPSS and YALE-2, respectively, p-value < 2 × 10–16). Positive correlation was confirmed by a non-parametric, one-sided Wilcoxon rank test (p-value pMPSS < 10–16, pYALE-2 < 10–16). YALE-1 was not significant (p-value pYALE-1~1.0).

## Discussion and conclusion

Rice genome projects have generated in depth genomic datasets and a comprehensive set of genomic upstream sequences. However, promoter sequences from other grass genomes have become available only sporadically. Comparative or computational biology approaches were therefore restricted to studies of individual pairs of interest and limited by the availability of only a few hundreds of grass promoter sequences. Our knowledge of *cis*-regulatory elements in monocotyledonous plants by the low number of known transcription factor binding sites is limited to those that have been reported and deposited in plant motif databases. The few dozens of known motifs are in sharp contrast to findings that higher plant genomes typically encode on average more than 1,500 transcription factors [36,37].

With the completion of the sorghum genome, a genome-wide assessment of regulatory sites in rice and sorghum upstream sequences has now become feasible. In this survey, we employed approaches based on two different tools, PhyloCon and FASTCOMPARE. Both tools and approaches have been successfully applied to motif discovery in many non-plant organisms including yeast and mammals. In addition, PhyloCon has previously been applied with success to *cis*-element analysis in genome survey sequences of *Brassica oleraceae *vs. *Arabidopsis thaliana *[13,18].

FASTCOMPARE is based on the 'network-level conservation' principle. This presupposes that regulatory circuitries will be largely conserved between two evolutionary related species and functional network motifs can be detected by their higher global or genome-wide conservation rate compared to non-functional sequences. Evolutionary conservation of functional elements is also assumed for phylogenetic footprinting that discovers motifs from a group of orthologous gene pairs. For the analysis based on PhyloCon, the orthologous groups that are compared and combined result from a prior selection of orthologous mate-pairs by co-expression analysis.

*Ab initio *analysis of *cis*-regulatory elements is notoriously error-prone due to small motif sizes and motif degeneracy. Our study was designed to select functional candidate sites and motifs that are associated with transcriptional activity. Co-expression was derived from correlations exceeding the top 1% of background similarities. Additionally, our clique approach required all group members to have a significant expression correlation with all other group members. In our survey, we restricted motif searches to 5'-upstream sequences of size 2 kb (for k-mer searches) or 3 kb (for PhyloCon) (i) to model current knowledge of plant promoter sizes and, (ii) to focus on plant core promoters that presumably contain most functional elements. Though functional enhancers and *cis*-elements in e.g. mammalian promoters, have been reported up to several tens of thousands bases distant to transcription initiation sites (TIS), plant promoters seem to be more compact [38]. In addition, chance co-occurrences will strongly increase, in particular, for smaller k-mers and degenerated motifs. Upstream sequences of larger size would thus have adverse effects by accumulating false positives or losing statistical power.

Results reported in this study can be divided into two categories: conserved sites and motifs. PhyloCon position specific scoring matrices (PSSMs) are supported by their conservation between orthologous promoters and their simultaneous co-occurrence in genes with expression similarities. Sizes of *cis*-elements in plants are comparable to non-plant species and typically range between 6 and 12 base pairs [5,6]. The mean size of PhyloCon PSSMs detected in this study was considerably longer (37 bp). Hence PSSMs likely represent concrete conserved sites rather than generalized statistical models for transcription factors. Large sizes for phylogenetic footprints in grasses are consistent with a previous study of 288 maize and rice pairwise and 56 rice, maize and sorghum three-way comparisons, in which a minimum motif size ≥ 20 bp was found to be significant [19]. Such long sites for PhyloCon PSSMs can be composed of two or more motifs and close proximity of these sites is required for functionality in the respective co-expressed group. Alternatively, some of the detected sites could represent signals associated with transcriptional gene activity such as mRNA stability signals or miRNA target sites, for which longer sizes have been reported [39]. Complementary to these long conserved regions, many of the detected network-level conserved motifs represent candidates for transcription factor binding sites. After subjecting the individual detected sites to clustering, in total 3,809 non-redundant motifs were found. The rice genome contains more than 1,600 genes encoding transcription factors and a similar number of *cis*-regulatory motifs could be expected [37]. However, some of our motifs may still be too specific and one transcription factor may bind to several related motifs. Consistent with this assumption, only for few k-mer positions did we observe sequence variability indicating that scoring functions favor specific k-mers or overrepresented k-mers with an overall low occurrence rate in a genome. Furthermore, many of these motifs were obtained from dyadic motif searches that converged to motifs with highly specified spacer sequences. For these long motifs, similar considerations may apply as for PhyloCon sites discussed above. Taking this into account, the number of motifs reported in this study is close to the number of transcription factors present in rice. On the other hand, our method may have missed transcription factor binding sites that tolerate high degeneracy. Similar findings for highly degenerated motifs have been reported for a FASTCOMPARE analysis in yeast [13]. Nevertheless, our list of motifs up to now provides the most comprehensive analysis of *cis*-elements in a grass genome.

In previous studies, the functionality of motifs has been confirmed by a variety of approaches. Many surveys have reported an association of motifs with particular biological processes. For large-scale analysis, gene ontologies or metabolic pathways were correlated with particular motifs. In this study, however, we were only able to detect a few such associations, and all enrichments were in very broad biological categories, e.g. 'transcription' (see Methods, results not shown). Missing associations likely result from limitations of the current rice GO annotation. In our search, we found for only 755 RAP2 rice genes (2.7%) at least one GO term belonging to the category 'biological process'. Similarly, only 1,376 rice genes (4.9%) could be mapped on KEGG pathways. In total, a functional annotation has been found for less than 5% of all rice genes. The sparse data basis and low resolution of the current rice GO annotation that mostly assigns top level terms, are the most probable causes for the limited success in detecting significant enrichments.

Several findings support the functionality of our motifs. PhyloCon sites are associated with conservation and co-expression. Despite the limited availability of experimentally verified *cis*-regulatory elements in grasses, we find numerous matches to known plant motifs or sites in public databases and literature reports. This includes many variants of the ACGT motif, like the G-box or the ABA response element as well as ethylene response elements among others. Interestingly, some top-scoring motifs do not match previously published elements and indicate novel *cis*-regulatory motifs. The number of motifs two rice genes has in common positively correlates with their expression similarity. This is consistent with the combinatorial nature of transcription regulation [40,35] and strongly indicates that a large fraction of detected motifs are associated with control of transcription. Control may be exerted as transcription factor binding sites or, as discussed previously, as miRNA target sites or signals for mRNA stability.

In summary, motifs reported in this study will provide researchers with a prioritized list of candidates for the gene of interest and can guide experimental designs for numerous sorghum and rice genes. Additional grass genome projects, for instance *Brachypodium distachyon*, a wheat relative, and maize are well advanced and can be expected to deliver important and information-rich comparative genome templates in the future [41]. This will enable and stimulate whole-genome comparative studies between three and more grass genome sequences. In particular, comparisons between two closely related grasses, maize and sorghum, will allow (i) branch-specific motifs to be accessed and, at the same time, (ii) the identification of motifs common to the monocot clade.

## Methods

### Determination of orthologous upstream sequences between rice and sorghum

Syntenic blocks between rice and sorghum have been described in [20]. To address possible complications caused by paralogs and tandem duplications, orthologs were selected by bidirectional best BlastP hits located in corresponding syntenic regions. To avoid misassignments caused by erroneous gene models, we restricted our analysis to gene pairs for which pairwise alignments included regions before the 15^th ^amino acid of either protein sequence. In total, 12,192 orthologous gene pairs were selected. Upstream sequences were defined as genomic sequences from the start codon to the start of the upstream preceding gene, with a maximal distance of 3 kb (PhyloCon analysis) and 2 kb (network-level conservation analysis). For sorghum we used gene models version 1.4 of [20], for rice the RAP2 gene annotation was used [25].

### Rice expression data processing

We used three different large-scale rice expression data sets, denoted as MPSS [23], YALE-1 [22] and YALE-2 [24].

The 70 mer oligonucleotides used on the YALE-1 and 2 arrays as well as the MPSS tag sequences were remapped to rice gene models of the RAP2 annotation. For both probe sets, only probes that unambiguously identify exactly one gene were used for the analysis. In cases in which no UTR information was available, for the respective transcript 100 bp of the respective 3'-downstream and 5'-upstream genomic sequence were added. For MPSS tags, we analyzed only the probe located nearest to the 3'-end of the transcript as this is expected to be the most informative [42,43]. In total, unambiguous mappings for 22,271 MPSS signatures and for 27,887 oligonucleotide probes were found and used for the subsequent analysis.

Filters were employed to identify signatures or oligonucleotide probes that were expressed abnormally rarely or could be generated by systematic errors. Starting from 22,271 mapped MPSS signatures, a total of 19,396 reliable and significant signatures were selected as described in [42,43]. YALE-1 data was normalized [22] and the strategy described in [44] was employed to derive a threshold for expressed probes. We determined an intensity cut-off of 410 with a 5% false positive rate (see additional file 9). In total, 13,904 rice genes/probes showed significant expression levels. For YALE-2, we adopted filter results from the original analysis resulting in 20,633 reliably expressed probes [23].

### Determination of co-expressed groups

For all expression datasets we determined background distributions of pairwise expression similarities from an all-against all Pearson correlation matrix. The 99%-quantile of these distributions was considered as significant for a pair to be defined as co-expressed. Thresholds r_min _for Pearson correlation coefficients were r = 0.79, r = 0.88, r = 0.93 for MPSS, YALE-1 and YALE-2, respectively (see additional file 10). In total, for 16,426, 13,223 and 18,820 distinct genes at least one co-expressed rice gene could be identified from the MPSS, YALE-1 and YALE-2 distributions respectively.

Networkx was used  to detect co-expressed gene groups in expression graphs. For an anchor gene its maximal clique was determined. We restricted our search to anchor genes with less than 100 edges. Identical cliques derived from different anchor genes were removed. Finally, 6,677 cliques covering 15,146 genes from MPSS data, 7,456 cliques including 11,412 genes from YALE-1 and 6,681 cliques comprising 8,793 genes for YALE-2 were retrieved.

### PhyloCon Motif discovery

To generate PhyloCon input data sets, only rice genes having an assigned ortholog in the 12,129 syntenic pairs rice-sorghum pairs were used. Cliques containing less than three pairs were discarded. The final MPSS, YALE-1 and YALE-2 datasets circumvented 4683, 4263 and 2185 cliques respectively, and included 6667, 4395 and 2379 rice genes, respectively.

*PhyloCon *was downloaded from [9]. Parameters for PhyloCon were selected as described previously [18]. Motif profiles reported by *PhyloCon *were transformed into PSSMs from alignments of all sorghum and rice instances. Transformation as well as statistical tests for motif overrepresentation under binomial distribution was performed as previously described [18]. P-values were adjusted for multiple hypotheses testing applying the Benjamini-Hochberg method [45] to correct for a false discovery rate of 5%. Overall 17068, 14754 and 5337 PSSMs were obtained from the MPSS, YALE-1 and -2 datasets, respectively. Simple repeats like motifs (GC)n were discarded.

Based on sequence similarities motif profiles were clustered. To estimate the similarity of two profiles, multiple sequence alignments of all instances were generated using *ClustalW *1.74 (gap opening penalty 1000 and gap extending penalty 0.001). The alignment score was the sum of column scores (match, mismatch and gap scoring 1,-1,-2, respectively) and normalized by size.

A similarity matrix based on all-against all pairwise PSSM alignments were constructed and hierarchical 'bottom up'-clustering was performed using the R package hclust. Clusters were determined by the cut-off corresponding to one-sided 5% significance deduced from the entire similarity matrix.

### Discovery of network-level conserved motifs

We followed the protocol of Elemento and Tavazoie [13]. We analyzed motif sizes ranging from 6 to 9-mers. Genome-wide conservation scores were calculated as ratios between observed numbers of co-occurrences in syntenic pairs versus the expected numbers. Transformation to z-scores allows the comparison of scores for motifs of different sizes.

Many transcription factors tolerate some degree of variability for particular site positions. Motifs searched for in this study were therefore represented as regular expressions and the (full) search space is represented by all possible subsets of the sequence space. We applied a heuristic approach to explore the search space. For each exact word, we randomly selected one position to which we added a randomly selected nucleotide. For each round of degeneration, we repeated this procedure four times. For each exact word, we analyzed 100 independent iterations.

Many significant motifs represent size (or regular expression) variations of one common motif theme or may be reported as significant because a subword/-string is significant. We defined a motif to be derived from another motif if it constitutes a (sub-)word of this motif. For variable positions, both motifs had to have overlapping specifities. To reduce redundancy, we ordered motifs according to their score, and removed all motifs from this list that were derived from a higher scoring motif.

We investigated dyad motifs with patterns of type {X}_a_{N}_b_{X}_a_, where X represents a specific letter, N represents any letter from the nucleotide alphabet, and a and b range from 2 to 4 and 4 to 12, respectively. A greedy scheme was applied to test whether more specified versions of the initially unspecific spacer sequence results in a higher scoring motif. For each position in the spacer, we determined the highest scoring representation of all 15 subset variations of the nucleotide alphabet, for instance {A},{AC},{AG} and so on. Next, we replaced each spacer position by its locally highest scoring letter representation starting from the position with the highest score improvement to the second, third and so on improvements. Motifs were re-scored after each replacement. Iterations were repeated as long as the total score of the motif increased.

### Validations

All statistical tests as well as multiple hypothesis testing corrections were carried out using R routines implemented in the R package stats.

The rice gene ontology (GO) categories were downloaded from the GO database (, 2008) and metabolic and molecular interaction pathways were identified in the KEGG database . Due to different rice annotation versions between these databases and the gene set used in this study, protein sequences corresponding to GO and/or KEGG pathways were remapped to our RAP2 cDNAs by either TBLASTN or BLASTN.

Comparisons against experimentally reported TFBSs were undertaken against all sites from the TRANSFAC (TRANSFAC 7.0, 2005, ) and PLACE databases (PLACE 30.0, 2007, ). In total, 96 non-redundant known rice sites were extracted from both databases. A multiple alignment between a known site and all sites included in the corresponding profile was generated and the alignment score was estimated as described above. For network-level conserved k-mers, string pattern matching was employed.

## Authors' contributions

XW and GH carried out the bioinformatic analysis and drafted the manuscript. GH and KFXM conceived the study, and participated in its design and coordination. All authors read and approved the final manuscript.

## Supplementary Material

Additional file 1 **Motif sites detected by PhyloCon in rice derived from MPSS**. Table showes motif sites detected by PhyloCon in rice genes derived from MPSS. The position of sites shown in the third column indicates the distance to the start codon. Click here for file

Additional file 2 **Motif sites detected by PhyloCon in rice derived from YALE-1**. Table showes motif sites detected by PhyloCon in rice genes derived from YALE-1. The position of sites shown in the third column indicates the distance to the start codon. Click here for file

Additional file 3 **Motif sites detected by PhyloCon in rice derived from YALE-2**. Table showes motif sites detected by PhyloCon in rice genes derived from YALE-2. The position of sites shown in the third column indicates the distance to the start codon. Click here for file

Additional file 4 **Motif sites detected by PhyloCon in sorghum derived from MPSS**. Table showes motif sites detected by PhyloCon in sorghum genes derived from MPSS. The position of sites shown in the third column indicates the distance to the start codon. Click here for file

Additional file 5 **Motif sites detected by PhyloCon in sorghum derived from YALE-1**. Table showes motif sites detected by PhyloCon in sorghum genes derived from YALE-1. The position of sites shown in the third column indicates the distance to the start codon. Click here for file

Additional file 6 **Motif sites detected by PhyloCon in sorghum derived from YALE-2**. Table showes motif sites detected by PhyloCon in sorghum genes derived from YALE-2. The position of sites shown in the third column indicates the distance to the start codon. Click here for file

Additional file 7 **Long motifs detected by network-level conservation analysis**. Table gives long motifs detected by network-level conservation analysis. The number of genes in rice and sorghum containing the respective motif in their upstream sequence are listed in the columns '#rice' and '#sorghum', respectively. Observed co-occurrences in syntenic pairs as well as the z-score are given. Click here for file

Additional file 8 **k-mer motifs detected by network-level conservation analysis**. Table gives k-mer motifs detected by network-level conservation analysis. The number of genes in rice and sorghum containing the respective motif in their upstream sequence are listed in the columns '#rice' and '#sorghum', respectively. Observed co-occurrences in syntenic pairs as well as the z-score are given. Click here for file

Additional file 9 **Determination of significant and reliable expression levels in YALE-1**. Significantly expressed probes have been determined according to [44]. Background expression is derived from all measurements of 58,404 oligonucleotide probes in 42 experiments. For each expression intensity, the percentage of measurements exceeding the respective expression level has been determined for two classes: (i) probes for which two or more replicates fell below the respective threshold and (ii) probes having higher intensities for 3 or more replicates. The x-axis depicts expression levels measured as the intensity of Cy5 dye, the y-axis the percentage of total measurements. For YALE-1, we found an expression intensity of 410 corresponding to the top 5% of all measurements. Click here for file

Additional file 10 **Background distribution for MPSS expression data**. Pearson correlations have been calculated for each gene versus all other genes. The correlation matrix has been used as background distribution for genome-wide expression similarities. The 99%-quantile has been numerically determined as significance level for co-expression of a gene pair. As an example, additional file 2 shows the background distribution for the MPSS expression data. X-axis depicts Pearson Correlation Coefficients, y-axis the number of gene pairs. The line marks the obtained 99%-quantile for MPSS at r = 0.79. Click here for file
