# Supplementary figures and images for: Discovery of cis-elements between sorghum and rice using co-expression and evolutionary conservation
Source: BMC Genomics. 2009 Jun 26;10:284. doi: 10.1186/1471-2164-10-284 (PMC2714861; doi:10.1186/1471-2164-10-284)

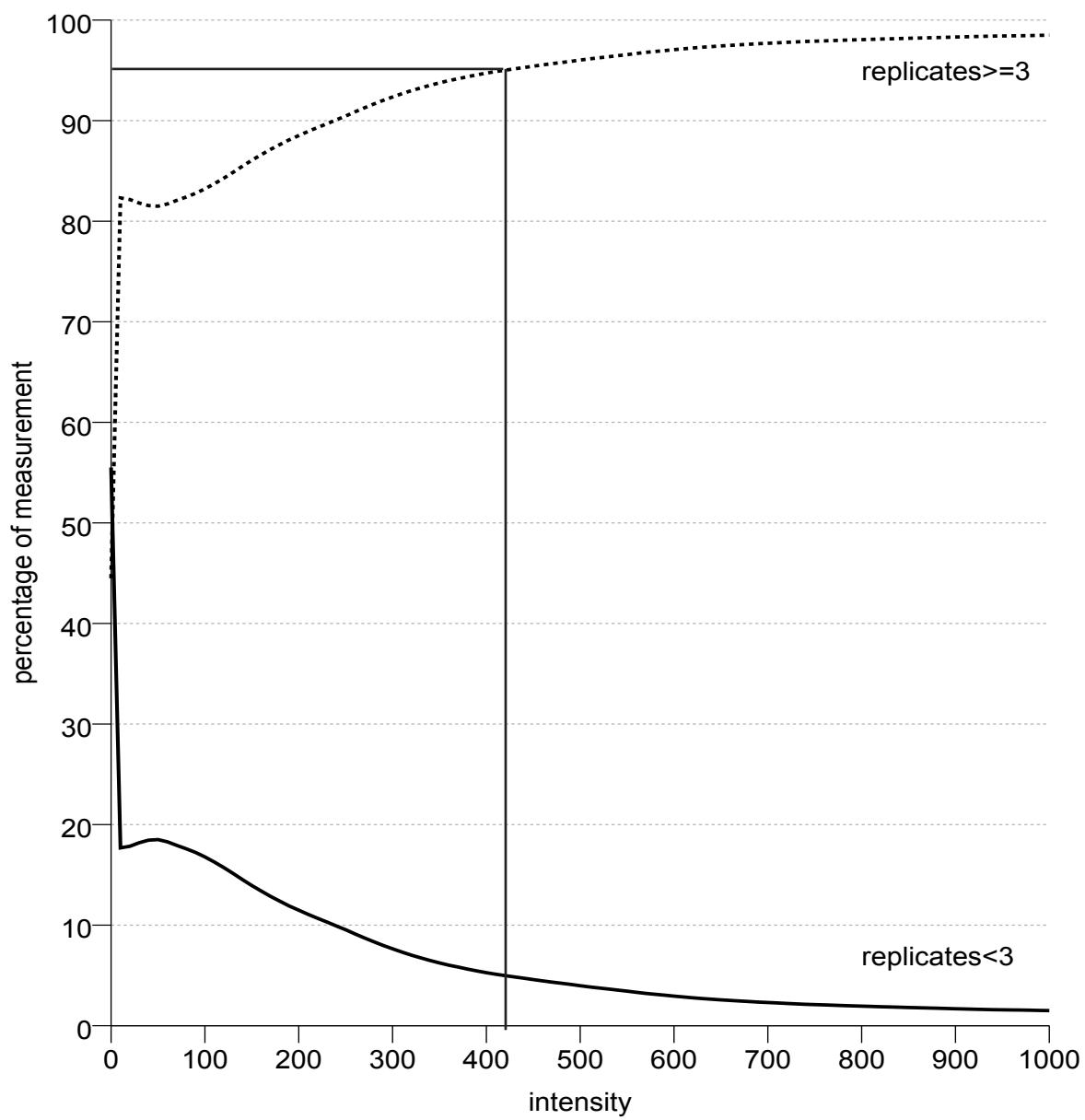

Supplement: Additional file 9 — Determination of significant and reliable expression levels in YALE-1. Significantly expressed probes have been determined according to [44]. Background expression is derived from all measurements of 58,404 oligonucleotide probes in 42 experiments. For each expression intensity, the percentage of measurements exceeding the respective expression level has been determined for two classes: (i) probes for which two or more replicates fell below the respective threshold and (ii) probes having higher intensities for 3 or more replicates. The x-axis depicts expression levels measured as the intensity of Cy5 dye, the y-axis the percentage of total measurements. For YALE-1, we found an expression intensity of 410 corresponding to the top 5% of all measurements. [file 1471-2164-10-284-S9.pdf]

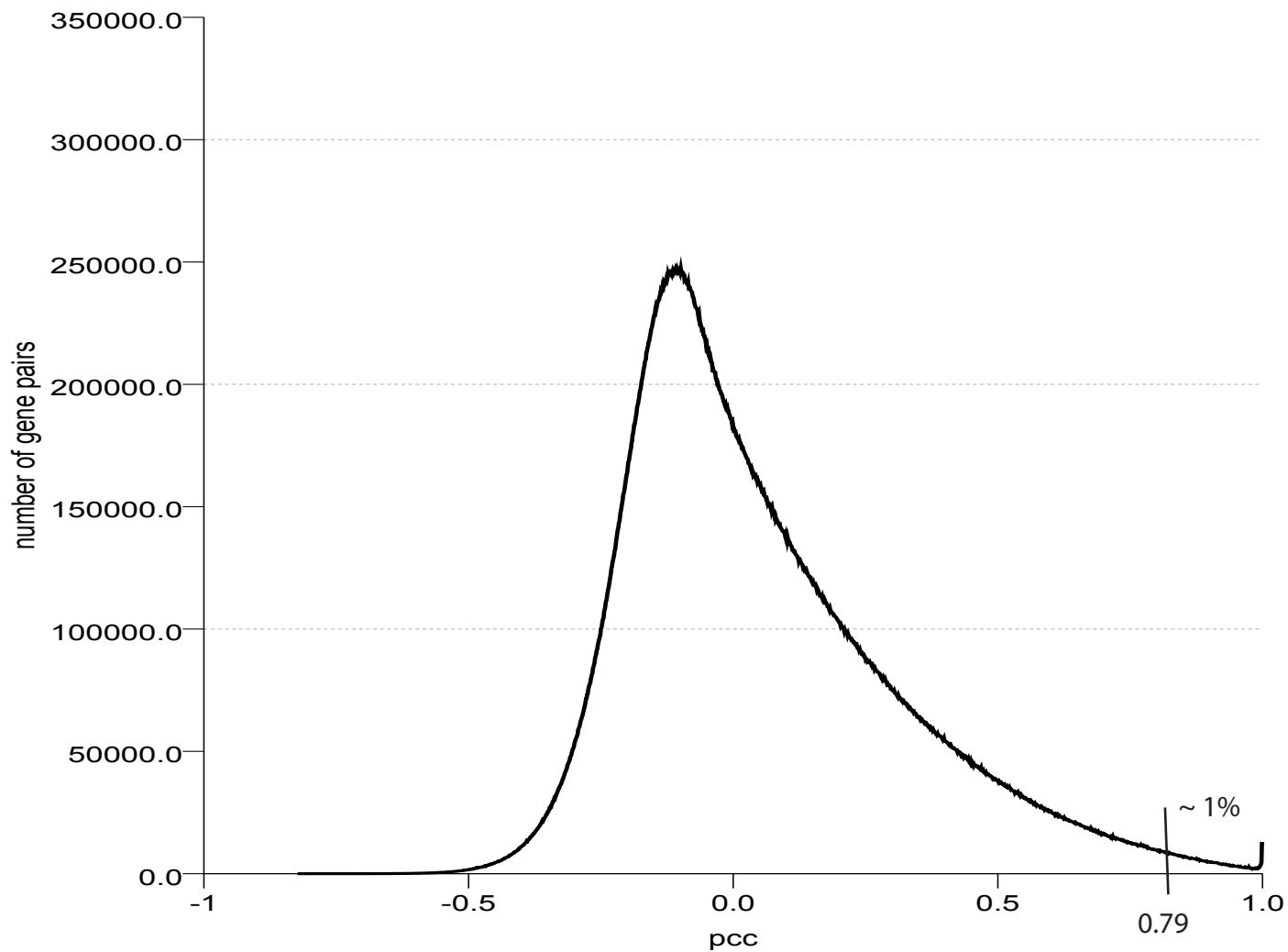

Supplement: Additional file 10 — Background distribution for MPSS expression data. Pearson correlations have been calculated for each gene versus all other genes. The correlation matrix has been used as background distribution for genome-wide expression similarities. The 99%-quantile has been numerically determined as significance level for co-expression of a gene pair. As an example, additional file 2 shows the background distribution for the MPSS expression data. X-axis depicts Pearson Correlation Coefficients, y-axis the number of gene pairs. The line marks the obtained 99%-quantile for MPSS at r = 0.79. [file 1471-2164-10-284-S10.pdf]
